# Intensive care unit acquired infection has no impact on long-term survival or quality of life: a prospective cohort study

**DOI:** 10.1186/cc5718

**Published:** 2007-03-09

**Authors:** Pekka Ylipalosaari, Tero I Ala-Kokko, Jouko Laurila, Pasi Ohtonen, Hannu Syrjälä

**Affiliations:** 1 Department of Infection Control, Oulu University Hospital, P.O. Box 22, FIN-90029 OYS, Finland; 2 Department of Anesthesiology, Division of Intensive Care, Oulu University Hospital, P.O. Box 22, FIN-90029 OYS, Finland; 3 Departments of Anesthesiology and Surgery, Oulu University Hospital, P.O. Box 22, FIN-90029 OYS, Finland

## Abstract

**Introduction:**

The aim of this study was to evaluate the impact of intensive care unit (ICU)-acquired infection on long-term survival and quality of life.

**Methods:**

Long-term survival was prospectively evaluated among hospital survivors who had stayed in a mixed, university-level ICU for longer than 48 hours during a 14-month study period during 2002 to 2003. Health-related quality of life was assessed using the five-dimensional EuroQol (EQ-5D) questionnaire in January 2005.

**Results:**

Of the 272 hospital survivors, 83 (30.5%) died after discharge during the follow-up period. The median follow-up time after hospital discharge was 22 months. Among patients without infection on admission, long-term mortality did not differ between patients who developed and those who did not develop an ICU-acquired infection (21.7% versus 26.9%; *P *= 0.41). Also, among patients with infection on admission, there was no difference in long-term mortality between patients who developed a superimposed (35.1%) and those who did not develop a superimposed (27.6%) ICU-acquired infection (*P *= 0.40). The EQ-5D response rate was 75 %. The patients who developed an ICU-acquired infection had significantly more problems with self-care (50%) than did those without an ICU-acquired infection (32%; *P *= 0.004), whereas multivariate analysis did not show ICU-acquired infection to be a significant risk factor for diminished self-care (odds ratio = 1.71, 95% confidence interval = 0.65–4.54; *P *= 0.28). General health status did not differ between those with and those without an ICU-acquired infection, as measured using the EuroQol visual-analogue scale (mean ± standard deviation EuroQol visual-analogue scale value: 60.2 ± 21 in patients without ICU-acquired infection versus 60.6 ± 22 in those with ICU-acquired infection). The current general level of health compared with status before ICU admission did not differ between the groups either. Only 36% of those employed resumed their previous jobs.

**Conclusion:**

ICU-acquired infection had no impact on long-term survival. The patients with ICU-acquired infection more frequently experienced problems with self-care than did those without ICU infection, but ICU-acquired infection was not a significant risk factor for diminished self-care in multivariate analysis.

## Introduction

Nosocomial infections increase mortality and costs in intensive care units (ICUs) [1-3]. Furthermore, they increase length of stay in the ICU as well as the frequency and duration of organ failures [4]. We previously showed that ICU-acquired infection was an independent risk factor for hospital mortality, even after adjustment for age and Acute Physiology and Chronic Health Evaluation (APACHE) II and Sequential Organ Failure Assessment (SOFA) scores, in a series of 335 ICU patients with ICU stay longer than 48 hours [5]. However, long-term outcome has not been studied in detail in patients acquiring an infection during their ICU stay. Studies have shown that sepsis patients who survive critical illness are at greater risk for post-ICU death than are control individuals; furthermore, survivors have been reported to have poor functional outcomes [6,7]. Reduced quality of life has also been reported among patients with acute respiratory distress syndrome (ARDS) as compared with critically ill control patients, but postdischarge mortality did not appear to be increased in ARDS patients [8,9]. ICU-acquired infections during critical illness impose a major burden on the costs and outcomes of intensive care; we addressed the question of whether these infections also have an impact on long-term mortality and quality of life in a prospectively study conducted in a subgroup of survivors after discharge.

## Materials and methods

### Study location and population

The study was conducted in Oulu University Hospital, which is a 900-bed tertiary level teaching hospital. All patients admitted into the ICU during the period from May 2002 to June 2003 whose ICU stay was longer than 48 hours were included in the study. The study protocol was approved by the hospital's ethics committee. The distribution of infections on admission and the epidemiology and contribution of ICU-acquired infections to hospital mortality were reported previously [5,10,11]. This substudy concentrated on the situation following hospital discharge.

### Study parameters

For all study patients the following information was collected: age, sex, smoking habits, alcohol abuse, presence of chronic underlying diseases (chronic obstructive pulmonary disease, ischaemic heart disease, chronic hepatic disease, chronic renal disease, previous stroke or transient ischaemic attack, diabetes, malignancy or immunosuppressive medication), severity of underlying diseases and organ dysfunctions on admission (assessed using APACHE II [12] and SOFA [13]), and diagnostic category on admission.

The presence of infection was recorded using criteria required by the US Centers for Disease Control and Prevention (CDC) [14,15] but with the following modifications. A catheter-related infection was deemed to be present if the same strains of bacteria were isolated in blood cultures and in a semiquantitative catheter tip culture, with no other site of infection. A catheter-related infection was also diagnosed if the patient had a positive semiquantitative catheter tip culture while blood cultures showed no growth or were not done, and there were clinical signs of infection, no other infection site was present and the patient exhibited a favourable response to antimicrobial therapy. Secondary bacteraemia was recorded when the same strains of bacteria were isolated in blood culture and in culture from a site of infection. Ventilator-associated pneumonia was defined according to criteria proposed by an international panel [16]. Pneumonia was diagnosed when a new and persistent infiltrate that was not otherwise explained appeared on chest radiographs, along with the presence of any two of the following: fever (temperature > 38°C) or hypothermia (temperature < 36°C), leucocytosis (> 10 × 10^3^/mm^3^) or leucopenia (< 4.0 × 10^3^/mm^3^), and new purulent tracheal aspirate.

Lengths of stay in the ICU and at hospital were recorded. Postdischarge mortality data were obtained from the hospital database, which had been updated with data from Central Statistical Office of Finland on 25 January 2005.

### Measurement of health-related quality of life

Health-related quality of life (HRQOL) was measured using the five-dimensional EuroQol (EQ-5D) questionnaire, which has been described in detail elsewhere [17]. It has been recommended and widely used for measuring HRQOL in critical care [18-21]. In short, the questionnaire contains two parts: the EQ-5D self-classifier, a self-reported description of current health problems according to five items (mobility, self-care, usual activities [work, housework, family and leisure activities, and so on], pain/discomfort and anxiety/depression) each with three response alternatives (1 = no problems, 2 = moderate problems, 3 = severe problems). The second part is a visual-analogue scale (EQ-VAS) ranging from 0 (worst possible health state) to 100 (best possible health state), on which the patients rate their current health. A weighted health state index, the EuroQol 5D Index, based on the five dimensions and ranging from -0.11 ('worse than death') to 1 ('perfect health'), was also calculated [22].

All survivors were mailed the following materials in January 2005: a cover letter explaining the objectives of the study and requesting the patient's or their relatives' collaboration in completing the questionnaire; a copy of the EQ-5D questionnaire; and a form with accessory questions regarding each patient's subjective overall assessment of their health status compared with the situation before ICU treatment and their current employment status. If there was no initial response to the questionnaire, the patients were contacted by phone by a trained ICU study nurse, who repeated the questions on the phone exactly, according to the EQ-5D questionnaire, and asked the patient to answer 'yes' or 'no'.

### Data registration and statistical analysis

The data were entered into a SPSS database (SPSS Data Entry, version 2.0; SPSS Inc., Chicago, IL, USA). Summary statistics for continuous or ordinal variables are expressed as the median with the 25th to 75th percentiles or as the mean and standard deviation (SD). The multivariate Cox regression model was used to assess the impact of ICU infection on long-time survival, whereas the other parameters in the final model were selected on statistical grounds (*P *< 0.05). The log-linearity assumption of the continuous variables was checked by creating a design variable based on quartiles, and the assumption of proportional hazards was evaluated graphically by log-minus-log survival plots. Log-rank test results are presented for Kaplan-Meier survival curves. The impact of ICU-acquired infection on EQ-5D self-care (no problems or some problems) dimension was evaluated by logistic regression analysis. The linearity assumption of the continuous variable for age did not hold, and a dichotomous covariate at age 50 years (< 50 versus ≥ 50) was therefore created. Goodness-of-fit was evaluated using the Hosmer-Lemeshow test. The other variables entered into the multivariate Cox and logistic regression models in addition to ICU-acquired infection were the APACHE II score, chronic underlying disease, infection on ICU admission, sepsis, severe sepsis or septic shock on admission, community or hospital-acquired pneumonia on admission, admission diagnostic category (medical, surgical nontrauma, trauma, neurological), ICU length of stay, SOFA score on admission and on ICU discharge, and the normal face validity parameters of age, sex, smoking habits and alcohol abuse. No significant interactions or collinearities between ICU infection and the other parameters in either multivariate model were found. Further, nested models were compared using the likelihood ratio test to select the best model. Two-tailed *P *values are reported, and the analyses were performed by the SPSS (version 12.0.1; SPSS Inc.) software.

## Results

### Characteristics of intensive care unit admissions

**Figure 1:**
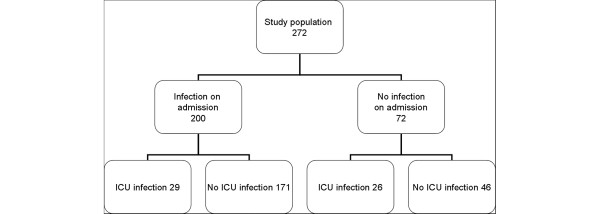
Study population. ICU, intensive care unit.

The study population is presented in Figure 1. The main demographic data and clinical characteristics of 272 patients discharged from hospital are presented in Table 1. There were significantly more patients with trauma on admission among the patients who developed an ICU infection (*P *< 0.001) and more medical admissions among the patients who did not develop an ICU infection (*P *< 0.001). The median APACHE II scores did not differ between the groups, whereas the median SOFA score on admission was higher and the ICU length of stay longer in the group of surviving patients with an ICU-acquired infection. The following ICU-acquired infections were recorded in 55 patients: ventilator-associated pneumonia (17), surgical site infections (14), lower respiratory tract infection (14), intra-abdominal infections (6), sinusitis (6), soft tissue or skin infections (4), primary or catheter-associated bacteraemia (2), secondary bacteraemia (1), urinary tract infection (1) and other infections (2).

**Table 1:** Main demographic data and clinical characteristics of patients discharged from hospital

Factor	No ICU-acquired infection (*n *= 217)	ICU-acquired infection (*n *= 55)	*P*
Male sex	133 (61.3)	40 (72.7)	0.12
Age years	57 (46–68)	57 (45.5–67.5)	0.63
Main reason for admission			
Surgical, nontrauma	73 (34.0)	25 (45.5)	0.12
Trauma	18 (8.4)	16 (29.1)	< 0.001
Medical	96 (44.7)	8 (14.5)	< 0.001
Neurological	21 (9.8)	4 (7.3)	0.8
Chronic underlying disease	148 (68.2)	33 (60.0)	0.27
Current smoker	82 (42.7)	20 (40.8)	0.87
Alcohol abuse	42 (19.4)	11 (20.8)	0.85
APACHE II score on admission	22 (18–28)	20 (17–27)	0.44
SOFA score on admission	6.0 (4.0–8.0)	9.0 (6.0–10.0)	< 0.001
SOFA score on ICU discharge	3.0 (2.0–5.0)	3.0 (2.0–4.0)	0.26
Infection on admission	171 (78.8)	29 (52.7)	< 0.001
Community-acquired pneumonia on admission	59 (27.2)	5 (9.1)	0.004
Hospital-acquired pneumonia on admission	32 (14.7)	7 (12.7)	0.83
Sepsis on admission	30 (13.8)	3 (5.5)	0.11
Severe sepsis on admission	17 (7.8)	0 (0)	0.03
Septic shock on admission	37 (17.1)	16 (29.1)	0.06
LOS in ICU (days)	3.75 (2.7–5.8)	10 (6.2–15.8)	< 0.001
LOS in hospital, days	17 (10–26)	25 (17.5–37)	0.62

Values are expressed as median (25th to 75th percentile) or number (%) of patients. Chronic underlying diseases included chronic obstructive pulmonary disease, ischaemic heart disease, chronic hepatic disease, chronic renal disease, previous stroke or transient ischaemic attack, diabetes, malignancy or immunosuppressive medication. APACHE, Acute Physiology and Chronic Health Evaluation; ICU, intensive care unit; LOS, length of stay; SOFA, Sequential Organ Failure Assessment.

### Outcomes

**Figure 2:**
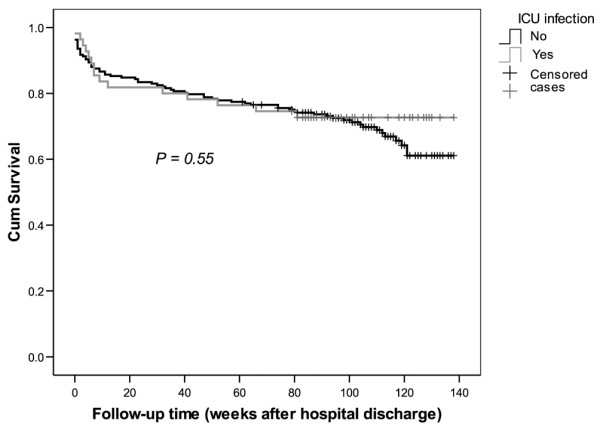
Survival curves of ICU patients after discharge from hospital. The patients who were alive on 25 January 2005 were censored. ICU, intensive care unit.

Of the 272 patients discharged from hospital, 85 died after discharge (31.3 %). The median follow-up time after hospital discharge for the whole study population was 22 months (25th to 75th percentile: 16 to 26 months) and that for the survivors was 24 months (21 to 28 months). This was also the median time for completing the EQ-5D questionnaire form. Among the patients with no infection on admission, seven patients with an ICU infection (26.9%) and 10 patients without an infection (21.7%; *P *= 0.41) died. The corresponding numbers of deaths among the patients with infection on admission were as follows: six patients with an ICU infection (27.6%) and 60 patients without an ICU infection (35.1%; *P *= 0.40). The long-term survival curves did not differ between the patients with and those without an ICU-acquired infection (Figure 2). Furthermore, based on the multivariate Cox model, ICU-acquired infection did not increase long-term mortality (Table 2). The adjusted hazard ratio for the effect of ICU-acquired infection on posthospital mortality in the multivariable Cox regression model was 0.83 (95% confidence interval [CI] 0.47–1.46).

**Table 2:** Effect of ICU-acquired infection on posthospital mortality in multivariable Cox regression model

Factor	HR	95% CI	*P*
ICU-acquired infection	0.83	0.47–1.46	0.52
APACHE II score on admission	1.07	1.04–1.46	< 0.001
Presence of chronic disease	3.23	1.6–6.54	0.001
Hospital-acquired pneumonia	2.6	1.57–4.33	< 0.001
Surgical reason for admission	1.93	1.23–3.03	0.004

APACHE, Acute Physiology and Chronic Health Evaluation; CI, confidence interval; HR, hazard ratio; ICU, intensive care unit.

### Health-related quality of life

Quality of life data were obtained from 142 of the 187 survivors (75.9%). The questionnaire was completed by the patient in 121 cases (85.2%), by relatives in 12 cases (8.5%), and by a district or ward nurse in nine cases (6.3%). A total of 113 respondents returned the questionnaire by mail, and 29 were contacted by phone. The nonrespondents were significantly younger than the respondents, but no significant differences were observed in sex, APACHE II scores, admission diagnostic category, presence of chronic underlying disease, or length of stay in the ICU or in hospital (Table 3). Only 36% of those employed before the ICU episode (*n *= 47) had resumed their previous jobs, whereas 54.4% had quit because of the illness that led to ICU admission.

**Table 3:** Main demographic data and clinical characteristics in respondents and nonrespondents to the EQ-5D questionairre

Factor	Nonrespondents	Respondents	*P*
Age (years)	48 (33–59)	57 (43–69)	0.008
Sex (male)	64.4%	61.3%	0.73
APACHE II score	23 (17–29)	20 (17–26)	0.19
Admission diagnostic category			
Trauma	22.2%	12.8%	0.15
Surgical, nontrauma	17.8%	33.3%	0.06
Medical	42.2%	40.4%	0.86
Neurological	15.6%	7.8%	0.15
Presence of chronic underlying disease	44.4%	59.9%	0.09
ICU length of stay (days)	4.7 (3.0–7.0)	4.4 (2.8–6.6)	0.82
Hospital length of stay (days)	14 (8–22)	18.5 (11–29)	0.05

Values are expressed as median (25th to 75th percentile) or number (%) of patients. APACHE Acute Physiology and Chronic Health Evaluation; EQ-5D, five-dimensional EuroQol; ICU, intensive care unit.

The EQ-5D self-classifier data of the study groups with and without an ICU-acquired infection are presented in Table 4. The patients with an ICU infection had more problems on the self-care dimension (*P *= 0.004), but there were no other differences. However, multivariate analysis did not identify ICU-acquired infection to be a risk factor for diminished self-care (odds ratio = 1.71, 95% CI 0.65 to 4.54; *P *= 0.28; Table 5). Overall, two-thirds of the patients suffered from moderate or extreme pain.

**Table 4:** EQ-5D results in long-term survivors

Parameter	All respondents	No ICU-acquired infection (*n *= 112/147^a^)	ICU-acquired infection (*n *= 30/40^a^)	Difference in proportions (95% CI)	*P*
Mobility					0.67
No problems	56 (39.4)	46 (41.1)	10 (33.3)	7.7 (-12.1 to +24.6)	
Some problems	74 (52.1)	56 (50.0)	18 (60.0)	-10.0 (-27.9 to +9.9)	
Confined to bed	12 (8.5)	10 (8.9)	2 (6.7)	2.3 (-12.9 to +10.5)	
Self-care					0.004
Complete	91 (64.1)	76 (67.9)	15 (50.0)	17.9 (-1.3 to +36.5)	
Some problems in washing or dressing	34 (23.9)	20 (17.9)	14 (46.7)	-28.8 (-47.0 to -10.5)	
Unable to wash or dress	17 (12)	16 (14.3)	1 (3.3)	11.0 (-3.4 to +19.1)	
Usual activities					0.32
No problems	59 (41.5)	50 (44.6)	9 (30.0)	14.6 (-5.3 to +30.9)	
Some problems	60 (42.3)	44 (39.3)	16 (53.3)	-14.0 (-32.6 to +5.5)	
Unable to perform	23 (16.2)	18 (16.1)	5 (16.7)	-0.6 (-18.4 to +11.6)	
Pain or discomfort					0.85
Not at all	48 (34.8)	38 (34.9)	10 (34.5)	0.4 (-19.6 to +17.7)	
Moderate	80 (58.0)	62 (56.9)	18 (62.1)	-5.2 (-23.1 to +15.0)	
Extreme	10 (7.2)	9 (8.3)	1 (3.4)	4.8 (-9.4 to +12.1)	
Anxiety or depression					0.91
Not at all	96 (70.6)	74 (69.2)	22 (75.9)	-6.7 (-21.8 to +13.0)	
Moderate	37 (27.2)	30 (28)	7 (24.1)	3.9 (-15.6 to +18.9)	
Extreme	3 (2.2)	3 (2.8)	0 (0)	2.8 (-9.0 to +7.9)	

Values are presented as number (percentage) of patients. ^a^Respondents/all long-term survivors. CI, confidence interval; EQ-5D, EuroQol five-dimensional questionnaire; ICU, intensive care unit.

**Table 5:** Effect of ICU-acquired infection on diminished self-care in multivariate logistic regression analysis

Factor	OR	95% CI	*P*
ICU-acquired infection	1.71	0.65–4.54	0.28
Hospital-acquired pneumonia	4.31	0.89–20.88	0.07
Age (≥ 50 versus <50 years)	2.75	1.04–7.29	0.04
Current smoker	0.35	0.13–0.91	0.03
Community-acquired pneumonia	0.27	0.08–0.89	0.03

-2 Log likelihood 137.700, P (Hosmer and Lemeshow test) = 0.898. CI, confidence interval; ICU, intensive care unit; OR, odds ratio.

The mean ± SD value on the EQ-VAS was 60.2 ± 21 among the patients without an ICU-acquired infection, and the corresponding figure for those with an ICU-acquired infection was 60.6 ± 22. The difference between the means was -0.41 (95% confidence interval = -9.30 to +8.48; *P *> 0.9). The mean ± SD EQ-5D weighted health state index (EuroQol 5D Index) was 0.715 ± 0.24 for those without an ICU infection and 0.725 ± 0.23 for those with an ICU infection. The difference between the means was -0.01 (95% CI = -0.11 to +0.09; *P *= 0.84).

The current general level of health compared with the status before ICU admission did not differ between the groups, because 52 (47.7%) of patients without and 16 (57.1%) of those with an ICU-acquired infection perceived their health status to be worse (*P *= 0.40). The difference between proportions was -9.4% (95% CI = -28.2% to +10.9%).

## Discussion

Our results show that ICU-acquired infection did not have significant impact on long-term mortality after discharge. Although the patients with an ICU-acquired infection more frequently experienced problems in self-care (50%) than did those without an ICU infection (33.2%; according to EQ-5D), ICU-acquired infection was not a significant risk factor for diminished self-care in multivariate analysis.

To date, there have been no previous studies looking at long-term outcomes of patients with ICU-acquired infections as a whole. Most of the published studies deal with long-term mortality from specific infections, often ones acquired before the ICU admission [6,23-25]. The strengths of our study are the prospective design and the systematic search for various infections on admission and during the ICU stay. The response rate of 75 % for the HRQOL survey was also in accordance with earlier studies using a similar survey method [21,26,27]. Because the nonrespondents were younger and exhibited a trend toward shorter hospital LOS, it is possible that our HRQOL results would have been better if the nonrespondents had also answered. Also, the time frame of more than 48 hours of ICU stay, which is generally used in association with ICU-acquired infections, may result selection bias in favour of very seriously ill patients and overestimation of mortality, as well as underestimation of the results of the HRQOL survey compared with whole ICU populations.

Some limitations of our study should be specifically addressed. Because the study was conducted in a single mixed ICU, there were not enough patients to evaluate the impacts of specific infections on outcome, which should be evaluated in a larger multicentre study. There are several possible explanations for the finding that an ICU-acquired infection did not appear to have an impact on HRQOL or post-hospital mortality. Our study population consisted of 272 patients, which was reflected in the wide 95% CIs. We observed, for example, a 9.4% difference in the proportions of the variable 'General health compared with pre-ICU situation (worse versus better/similar)' between the two groups. According to power calculations (with α = 0.05 and β = 0.20, and assuming that 25% of the patients actually had an ICU-acquired infection), we would have needed approximately 1,250 patients to prove that the difference was statistically significant. This would have required nine years of data collection with our study protocol in this centre. Another explanation may be the fairly long follow-up time. However, there were no significant differences in median follow-up times (21 months for the patients with and 23 months for those without an ICU infection; *P *= 0.57), which is an argument against this explanation. The third, and likely, explanation is that the ICU-acquired infection really did not have any impact on posthospital mortality or HRQOL.

The present study shows that ICU-acquired infection did not increase long-term mortality after hospital discharge, suggesting that the patients with serious ICU-acquired infections who are likely to die actually die during their hospital stay; ICU-acquired infection has been shown to be an independent risk factor for hospital mortality [5]. Similar results were previously reported for ARDS patients [8]. According to our risk model, known factors such as severity of illness, presence of chronic underlying disease, and admission category were risk factors for long-term mortality even in our series [28-30]. Although hospital-acquired pneumonia on admission to the ICU was not a risk factor for hospital mortality, it was a significant risk factor for long-term mortality among the patients surviving hospital discharge. This was most likely due to the more severe underlying diseases in this patient group [10]. Needless to say, ICU-acquired infection would not have been entered into the Cox regression model if we had been studying overall long-term mortality among ICU survivors.

Although in our series the majority of respondents reported moderate or good general health after discharge, almost half of them rated their health as worse than before ICU admission. Quality of life was generally reduced to the same extent in patients with and those without an ICU-acquired infection, as measured using the EQ-VAS and the EuroQol 5D Index. There was, however, a difference in one of the dimensions of the EQ-5D self-classifier. Namely, patients with an ICU-acquired infection experienced more problems on the self-care dimension than did those without an ICU-acquired infection, but ICU-acquired infection was not a significant risk factor for diminished self-care in multivariate analysis. It has previously been shown that follow up-HRQOL reflects preadmission HRQOL [31]. The rate of decline of HRQOL was higher in our series than in a German study [32] in which the physical and mental components deteriorated in only 14% and 8% of the survivors, respectively. The fact that we did not rate the preadmission HRQOL might have had an influence on the patient's perception of the change in HRQOL. However, a baseline assessment was not possible in our patient series. Furthermore, it is known that proxies underestimate patients' quality of life [33-35]. In addition to the preadmission HRQOL, the underlying comorbidities, the degree of organ dysfunction, and the length of ICU stay might also have influenced the long-term quality of life. Accordingly, more than 60% of our patients had a chronic underlying disease, which has been shown to have a significant effect on post-ICU HRQOL [26]. The degree of organ dysfunction has been shown to be related to the diminished quality of life [32]. Although our patients with ICU-acquired infection had more severe organ dysfunctions on admission, there were no differences at ICU discharge. Some differences in HRQOL have been shown to exist between patients undergoing short-term and those undergoing long-term ICU stays with regard to physical role and vitality [36]. In our series, the patients with an ICU-acquired infection also had a significantly longer ICU stay. The rate of septic shock on admission was higher in the group with ICU-acquired infections, but patients with sepsis and septic shock have been previously shown to regain quality of life similar to that in other critically ill patients [19].

Overall, ICU-acquired infection was not a significant risk factor for diminished self-care in multivariate analysis. Age over 50 years was a risk factor for diminished self-care, whereas smoking and community-acquired pneumonia seemed to be protective factors. Because our aim was to study the significance of ICU-acquired infections for long-term quality of life, this variable was necessarily included in the different models. Hence, the odds ratios of the other parameters are biased because of the incorporation of ICU-acquired infection in the models. Therefore, any conclusions concerning the other parameters should be made with caution. The possibility of random effects in a small sample may naturally be one explanation. Despite their self-care problems, the patients who survived an ICU infection were equally likely to regain their prior general health status as were ICU patients without an ICU-acquired infection.

Two-thirds of the present patients suffered from moderate or extreme pain, supporting the earlier findings that long-term pain and depression may persist even after patients have restored their physical capacity [21,27]. In addition, two-thirds of employed respondents had not resumed their previous jobs by the time of the questionnaire survey, mainly because of the illness that led to their ICU admission; the figure is similar to that earlier reported for ARDS survivors [37]. This may have a significant influence on families and society.

## Conclusion

ICU-acquired infection did not independently influence long-term survival or quality of life after hospital discharge in this series. Our results should be verified in a prospective multicentre study.

## Key messages

• ICU-acquired infection did not affect long-term mortality in patients surviving hospital discharge.

• Those surviving an ICU infection regained a similar quality of life, compared with their pre-ICU situation, as did those without an ICU infection.

• Patients with an ICU-acquired infection experienced more problems in self-care than did those without an ICU-acquired infection. However, ICU-acquired infection was not a significant risk factor for diminished self-care in multivariate analysis.

## Abbreviations

APACHE = Acute Physiology and Chronic Health Evaluation; ARDS = acute respiratory distress syndrome; CI = confidence interval; EQ-5D = EuroQol five-dimensional questionnaire; EQ-VAS = EuroQol visual-analogue scale; HRQOL = health-related quality of life; ICU = intensive care unit; SD = standard deviation; SOFA = Sequential Organ Failure Assessment.

## Competing interests

The authors declare that they have no competing interests.

## Authors' contributions

PY participated in the design of the study and the acquisition and analysis of data, and drafted the manuscript. TA-K, JL and HS participated in the design of the study and analysis of data, and drafted the manuscript. PO participated in the design of the study and performed the statistical analysis. All authors read and approved the final manuscript
